# Prevalence and associated factors of breastfeeding in women with gestational diabetes in a University Hospital in Thailand

**DOI:** 10.1186/s13006-019-0227-8

**Published:** 2019-07-31

**Authors:** Preeyaporn Jirakittidul, Nalinee Panichyawat, Benjaphorn Chotrungrote, Athitaya Mala

**Affiliations:** Family Planning and Reproductive Health Unit, Department of Obstetrics and Gynecology, Faculty of Medicine Siriraj Hospital, Mahidol University, Bangkok, Thailand

**Keywords:** Breastfeeding, Exclusive breastfeeding, Gestational diabetes mellitus, Prevalence, Postpartum

## Abstract

**Background:**

Gestational diabetes mellitus (GDM), which is a medical complication that develops during pregnancy, is associated with several long-term health problems. Despite several benefits of exclusive breastfeeding (EBF), including reduction in long-term health problems in mothers with GDM, few studies have investigated breastfeeding in women with GDM and information in the Thai population is lacking. The aim of the study was to determine the prevalence of breastfeeding and the factors associated with breastfeeding during the first six months postpartum in women with GDM.

**Methods:**

A questionnaire-based prospective study was conducted during November 2014 to June 2017. Study participants were first interviewed on the second day post-delivery, and then by telephone at 6 weeks, 3 months and 6 months postpartum. Breastfeeding assessment based on infant feeding practice in the last 24-h period was classified according to World Health Organization definitions.

**Results:**

A total 229 women were enrolled in this study. Prevalence of any breastfeeding at 24 h, 6 weeks, 3 months, and 6 months postpartum was 28.8% (*n* = 66), 94.3% (*n* = 214), 71% (*n* = 154), and 49.8% (*n* = 104), respectively. Prevalence of EBF was 35.9% (*n* = 78) at 3 months, and 23% (*n* = 48) at 6 months after delivery. Maternal intention to breastfeed for 6 months was an independent predictor for both 6 months EBF (RR 16.38; 95% CI 2.29, 116.99) and any breastfeeding (RR 2.65; 95% CI 1.65, 4.25). Breastfeeding initiation within 24 h postpartum (RR 1.38; 95% CI 1.08, 1.76) and being a government officer or private business owner (RR 1.66; 95% CI 1.03, 2.68) were independent predictors of any breastfeeding and EBF for 6 months, respectively.

**Conclusion:**

The prevalence of breastfeeding in Thai women with GDM was lower than the national and global target goal. Maternal intention to breastfeed for at least 6 months and breastfeeding initiation were important factors associated with 6 months’ breastfeeding. In order to improve the breastfeeding rate and duration, health care providers should support women’s feeding decision, emphasize the benefits of breastfeeding to enhance breastfeeding intention, seek to remove or minimize barriers to the initiation of breastfeeding and reduce mother-infant separation time.

## Background

Gestational diabetes mellitus (GDM) is a common medical complication that develops during pregnancy, with a pooled prevalence of 10.1% and substantial variations across nations [1]. GDM is defined as new onset or first evidence of abnormal glucose tolerance that occurs during gestation [2]. In addition to causing immediate adverse effects on pregnancy outcomes, GDM can also cause long-term health problems in both mother and child. Previous studies have reported increased risk of type 2 diabetes mellitus (T2DM), metabolic syndrome, and cardiovascular disease (CVD) later in life in women diagnosed with GDM [3–6]. An epidemiologic study reported increased risk of long-term adverse effects, such as T2DM, obesity, metabolic syndrome and other chronic non-communicable diseases in adulthood in children of mothers with GDM [7].

Several international strategies have been developed to strongly support and promote breastfeeding as an optimal infant feeding strategy to ensure that children receive appropriate nutrition for growth, development, and health. The World Health Organization (WHO) and the United Nations Children’s Fund (UNICEF) recommend exclusive breastfeeding (EBF) during the first six months of life, and the introduction of safe and nutritionally adequate complementary foods starting at 6 months, together with continued breastfeeding up to 2 years of age or beyond. In accordance with these recommendations, the Thai Ministry of Public Health implemented the National Breastfeeding Project in 1989 to promote EBF. The target compliance rates established by the 11th National Economic and Social Development Plan (NESDP) of Thailand 2012–2016 were 30% during 2012–2016, and 50% during 2017–2021. Despite the implementation of this initiative, the 6-month EBF rate from a national survey conducted in Thailand in 2015 was only 23.1% [8]. This rate was lower than the 30% goal, and was lower than the global EBF rate of 34.8% reported by the WHO [9].

Maternal benefits from breastfeeding include a reduction in the risk of developing hypertension, dyslipidemia, T2DM, CVD, breast cancer and ovarian cancer. Two studies reported that breastfeeding in mothers with GDM can decrease the risk of developing T2DM and metabolic syndrome later in life [10, 11]. Despite these advantages, some previous studies reported a lower rate and duration of breastfeeding in mothers with GDM compared to women without GDM [12–14]. Two previous studies reported the prevalence of any breastfeeding at 6 months postpartum among mothers with GDM, 83.9% in Vietnam [14] and 31.3% in the UK [15]. However, those reports lacked data relating to either breastfeeding characteristics or prevalence of exclusive breastfeeding. Data specific to breastfeeding in Thai women with GDM are still lacking, with the few studies that did report breastfeeding rates in general Thai populations noting that these were lower than the 30% target [8, 16, 17]. Increased knowledge about the prevalence of and factors associated with breastfeeding in this Thai subpopulation will help us understand the negative and positive factors that influence breastfeeding behavior.

Accordingly, the aim of the present study was to determine the prevalence of breastfeeding and to identify factors associated with breastfeeding in mothers with GDM during the first six months postpartum.

## Methods

This questionnaire-based prospective study was conducted at the Family Planning and Reproductive Health Unit, Department of Obstetrics and Gynecology, Faculty of Medicine Siriraj Hospital, Mahidol University, Bangkok, Thailand during the November 2014 to June 2017.

Eligibility criteria were early postpartum women aged 20 years and over who delivered a singleton neonate, had attended antenatal care at Siriraj Hospital and had been diagnosed as having GDM during the antenatal period by Carpenter and Cousil criteria for 100-g oral glucose tolerance test (OGTT) or 1-h 50-g glucose challenge test (GCT) ≥ 200 mg/dL. Individuals with contraindications to breastfeed were excluded at recruitment. During the study period, woman with one or more of the following were additionally excluded: diagnosis of T2DM at 6 weeks postpartum, inability to follow up 75-g OGTT testing at 6 weeks postpartum, and/or death of infant prior to the age of 6 months.

Women diagnosed with GDM received standard antenatal care, including antenatal follow-up schedule as appropriate, dietary recommendation and monitoring of fasting and 2-h postprandial blood glucose. Women also received standard care during the intrapartum phase and the mode of delivery was determined by obstetric indications. After birth, all infants were admitted to the nursery ward to monitor blood glucose and to receive early infant formula feed to prevent hypoglycemia. Infant’s lingual frenulum and mother’s nipple anatomy were routinely assessed, as described elsewhere [18]. The appointments for all women with GDM were scheduled at 6 weeks postpartum at the Family Planning and Reproductive Health Unit for routine postpartum checkup, family planning and contraceptive counseling, and for 75-g OGTT testing.

All enrolled participants were first interviewed on the second day post-delivery before hospital discharge to collect the following information: maternal baseline characteristics, obstetric history, intentions regarding breastfeeding, duration of maternity leave, breast-related problems, and infant-related problems. Subsequently, interviews were scheduled at 6 weeks, 3 months and 6 months postpartum. The follow-up interviews elicited information relative to breastfeeding type, duration of breastfeeding, and reason for breastfeeding discontinuation.

Breastfeeding was defined as an infant who received breast milk regardless of method of feeding. In terms of breastfeeding assessment, mothers were asked about infant feeding practices based on the last 24-h feeding period [19]. Breastfeeding types were classified according to WHO definitions, as follows: EBF was defined as an infant who received only breast milk; predominant breastfeeding was defined as an infant who received breast milk as the predominant source of feeding which might also include water or fruit juice; complementary breastfeeding was defined as an infant who received breast milk and solid food, semisolid food and/or non-human milk; and no breastfeeding was defined as an infant who did not receive breast milk.

### Sample size calculation and statistical analysis

The sample size was calculated using a 31.3% prevalence rate of any breastfeeding at 6 months postpartum in women with GDM from a previous study which compared breastfeeding rates in women with GDM and women with type 1 or type 2 diabetes in a UK population [15]. Using a significance level of 0.05 (2-sided) and an absolute precision error of 7%, a total of 169 women was calculated.

Statistical analysis was performed using Stata statistical software (version 14) (StataCorp LLC, College Station, TX, USA). Demographic and clinical data were summarized using descriptive statistics. Data were presented as mean ± standard deviation (SD) or number (n) and percentage. Chi-square test or Fisher’s exact test was used for comparing categorical data, and t-test or Mann-Whitney U test was used for comparing continuous data as appropriated. Binary regression analysis was performed to identify factors significantly associated with breastfeeding for 6 months. A *p*-value less than 0.05 was considered to be statistically significant.

## Results

Three hundred and ninety-eight postpartum Thai women with GDM were initially invited to participate in this study. Of those, 169 women were excluded from data analysis due to an abnormal result or no result of the 75-g OGTT testing at 6 weeks postpartum (*n* = 168) or early neonatal death (n = 1). The remaining 229 mothers with GDM were included. Baseline demographic and clinical characteristics of the 229 mothers with GDM and their infants are shown in Table 1. The mean age was 33.2 ± 5.1 years (range 20–47 years) and the mean body mass index was 24.5 ± 5.2 kg/m^2^ (range 15.56–44.08 kg/m^2^). The majority of participants were married (96.9%). Nearly half of women were primiparous (46.2%), graduated with a bachelor degree or higher (56.7%) and delivered by cesarean section (46.3%). The mean maternity leave length was 1.5 months and ranged from 0 to 3 months. Approximately 18% of participants had nipple inversion or retraction. Almost one-fifth (18.7%, 41/219) of infants had severe tongue tie and 38 of those (92.7%) underwent frenotomy. With regard to breastfeeding duration, 49 women breastfed a prior child for at least 6 months and 147 women expressed an intention to breastfeed their current babies for at least 6 months.

**Table 1:** Demographic and clinical characteristics of 229 mothers with gestational diabetes and their infants

Characteristics	Mean ± SD or *n* (%)
Age (years)	33.2 ± 5.1
Marital status	
Single	2 (0.9)
Married	222 (96.9)
Separated	5 (2.2)
Educational level (*n* = 224)	
Primary school	21 (9.4)
High school	76 (33.9)
Bachelor degree or higher	127 (56.7)
Occupation	
Housewife	54 (23.6)
Employee	128 (55.9)
Government officer	14 (6.1)
Private business owner	33 (14.4)
Maternity leave (months, *n* = 228)	
No limit (housewife)	54 (23.7)
No leave	49 (21.5)
1 months	3 (1.3)
1.5 months	9 (3.9)
2 months	13 (5.7)
3 months	100 (43.9)
Family earnings (THB/month)	
< 10,000	37 (16.2)
10,000-50,000	167 (72.9)
> 50,000	25 (10.9)
Primiparity	107 (46.2)
Pre-pregnancy body mass index	
Normal	139 (60.7)
Overweight	53 (23.1)
Obese	37 (16.2)
Pre-pregnancy body mass index (kg/m^2^)	24.5 ± 5.2
Gestational weight gain (kg, n = 228)	11.5 ± 4.9
Gestational age at delivery (weeks)	37.8 ± 2.2
Cesarean section delivery	106 (46.3)
Inversion or retraction of nipple (*n* = 227)	40 (17.6)
Infants with severe tongue tie (*n* = 219)	41 (18.7)
Completed 6-month EBF in prior infant (*n* = 87)	35 (40.2)
Completed 6-month any breastfeeding in prior infant (*n* = 79)	49 (62.0)
Intention time to breastfeed in index pregnancy (*n* = 214)	5.7 ± 3.1
Intention to breastfeed at least 6 months postpartum (*n* = 214)	147 (68.7)
Contraceptive method (*n* = 227)	
Non-hormonal contraception	107 (47.1)
Progestin-only contraception	106 (46.7)
Combined hormonal contraception	14 (6.2)

*SD* standard deviation, *THB* Thai baht, *EBF* exclusive breastfeeding

**Fig. 1:**
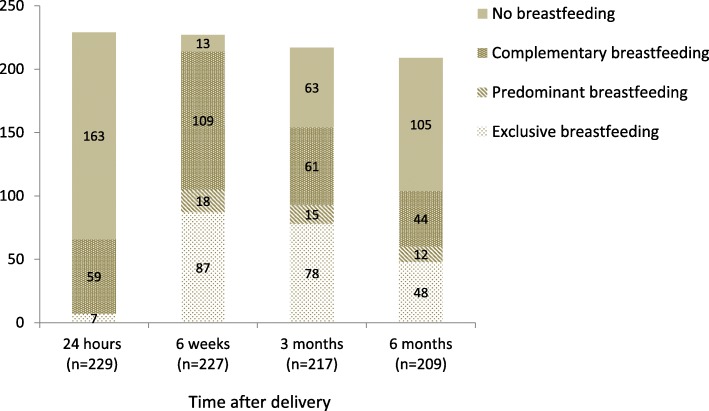
Prevalence of breastfeeding according to WHO definition

The prevalence of breastfeeding according to the WHO definition is shown in Fig. 1. Any breastfeeding rate at 24 h, 6 weeks, 3 months, and 6 months postpartum was 28.8, 94.3, 71.0, and 49.8%, respectively. Prevalence of EBF was 35.9% at 3 months and 23.0% at 6 months after delivery. The reasons cited for discontinuation of any breastfeeding before 6 months postpartum were self-perception of insufficient breast milk quantity (53.5%, 69/129), end of maternity leave (34.1%, 44/129), early infant feeding with semi-solid food (11.6%, 15/129) and a sick baby (0.8%, 1/129).

Factors associated with discontinuation of EBF and any breastfeeding prior to 6-months postpartum are shown in Table 2. Educational level, occupation, cesarean delivery, a reported intention to breastfeed for a full 6-months postpartum, and intended time to breastfeed in the index pregnancy were significantly associated with rates of 6-month EBF practice. Length of maternity leave, inverted/retracted nipple, a reported intention to breastfeed for a full 6-months postpartum, intention time to breastfeed in index pregnancy, initiation breastfeeding within 24 h after delivery, and contraceptive method were significantly associated with any breastfeeding for 6 months postpartum. Bachelor degree or higher educational level, working as a private business owner or government officer, vaginal delivery and an intention to breastfeed for 6-months postpartum in the index pregnancy were significantly associated with a higher rate of 6-month EBF practice. Reporting an intention to breastfeed for 6-months postpartum in the index pregnancy or initiation of breastfeeding within 24 h after delivery were significantly associated with a higher rate of any breastfeeding for 6 months postpartum. On the contrary, women with inverted/retracted nipple(s) were associated with a lower rate of any breastfeeding at 6 months postdelivery.

**Table 2:** Comparison of characteristics associated with discontinuation of breastfeeding prior to 6-months postpartum in the exclusive breastfeeding and any breastfeeding groups (*N* = 209)

Factors	Exclusive breastfeeding		*p*-value	Any breastfeeding		*p*-value
	< 6mo *N* = 161	≥ 6mo *N* = 48		< 6mo *N* = 105	≥ 6mo *N* = 104	
	Mean ± SD or n (%)	Mean ± SD or n (%)		Mean ± SD or n (%)	Mean ± SD or n (%)	
Age (years)	33.7 ± 5.3	32.8 ± 4.0	0.201	33.5 ± 5.2	33.4 ± 4.8	0.951
Marital status			1.000			0.683
Single	2 (1.2)	0 (0)		1(0.9)	1 (1.0)	
Married	156 (96.9)	47 (97.9)		103(98.2)	101 (96.1)	
Separated	3 (1.9)	1 (2.1)		1(0.9)	3 (2.9)	
Educational level (*n* = 205)			0.001			0.166
Primary school	17 (10.8)	4 (8.5)		11 (10.7)	10 (9.8)	
High school	57 (36.0)	10 (21.3)		35 (34.0)	32 (31.4)	
Bachelor degree	84 (53.2)	28 (59.6)		57 (55.3)	55 (53.9)	
Master degree or higher	0 (0.0)	5 (10.6)		0 (0.0)	5 (4.9)	
Occupation (N = 209)			0.016			0.092
Housewife	41 (25.5)	9 (18.7)		24 (22.9)	26 (25.0)	
Employee	92 (57.1)	22 (45.7)		65 (61.9)	49 (47.1)	
Government officer	6 (3.7)	8 (16.7)		4 (3.8)	10 (9.6)	
Private business owner	22 (13.7)	9 (18.8)		12 (11.4)	19 (18.3)	
Maternity leave length for 3 months (*n* = 158)	66 (55.0)	23 (60.5)	0.579	48 (59.3)	41 (53.2)	0.521
Family earnings (THB/month)			0.212			0.180
< 10,000	29 (18.0)	7 (14.6)		17 (16.2)	19 (18.3)	
10,000-50,000	117 (72.7)	32 (66.7)		80 (76.2)	69 (66.3)	
> 50,000	15 (9.3)	9 (18.7)		8 (7.6)	16 (15.4)	
Primiparity	73 (45.3)	25 (52.1)	0.416	47 (44.8)	51 (49.0)	0.580
Pre-pregnancy body mass index (kg/m^2^)	24.4 ± 4.9	23.7 ± 5.9	0.439	24.9 ± 5.1	23.7 ± 5.0	0.088
Gestational weight gain	11.2 ± 4.8	12.4 ± 4.9	0.129	11.4 ± 5.1	11.5 ± 4.6	0.914
Gestational age at delivery	37.5 ± 2.2	38.1 ± 1.9	0.104	37.5 ± 2.1	37.8 ± 2.2	0.456
Cesarean section delivery	81 (50.3)	15 (31.3)	0.022	54 (51.4)	42 (40.4)	0.127
Inversion or retraction of nipple (n = 209)	31 (19.5)	4 (8.3)	0.081	24 (23.3)	11 (10.6)	0.016
Infants with severe tongue tie (*n* = 202)	28 (18.1)	10 (22.2)	0.334	15 (14.9)	23 (23.2)	0.092
History of completed 6-month EBF (*n* = 177)	20 (15.2)	12 (26.7)	0.115	12 (13.8)	20 (22.2)	0.173
History of completed 6-month any breastfeeding (n = 168)	29 (22.7)	13 (32.5)	0.216	15 (19.5)	27 (29.7)	0.154
Intention time to breastfeed in index pregnancy (months, n = 197)	5.2 ± 2.9	7.0 ± 3.3	< 0.001	4.6 ± 2.2	6.6 ± 3.5	< 0.001
Intention to breastfeed at least 6 months postpartum (*n* = 197)	91 (59.5)	43 (97.7)	< 0.001	52 (51.5)	82 (85.4)	< 0.001
Initiation of breastfeeding within the first 24 h after delivery (*n* = 204)	44 (27.3)	17 (35.4)	0.238	21 (20.0)	40 (38.5)	0.004
Contraceptive method			0.007			< 0.001
Non-hormonal contraception	81(50.3)	18 (37.5)		53 (50.5)	46 (44.2)	
Progestin-only contraception	66 (41.0)	30 (62.5)		39 (37.1)	57 (54.8)	
Combined hormonal contraception	14 (8.7)	0(0.0)		13 (12.4)	1 (1.0)	

*SD* standard deviation, *THB* Thai baht, *EBF* exclusive breastfeeding

A *p*-value < 0.05 indicates statistical significance

Univariate and multivariable analysis for factors that independently predicted 6 months postpartum of EBF or any breastfeeding are shown in Table 3. After adjustment for confounding factors, maternal intention to breastfeed until 6 months of infant age was an independent predictor of both 6 months of EBF and any breastfeeding practice. Initiation of breastfeeding within 24 h after delivery was an independent predictor of any breastfeeding for 6 months and having an occupation as a government officer or private business owner was an independent predictor of EBF for 6 months.

**Table 3:** Univariate and multivariable analysis of factors associated with 6-month exclusive breastfeeding and any breastfeeding

Exclusive breastfeeding	Crude RR (95% CI); *p*-value	Adjusted RR (95% CI); *p*-value
Bachelor degree or higher	1.77 (1.01, 3.10); 0.045	1.26 (0.74, 2.13); 0.387
Intention to breastfeed for at least 6 months postpartum	20.21 (2.84, 143.50); 0.003	16.38 (2.29, 116.99); 0.005
Cesarean section delivery	0.53 (0.30, 0.92); 0.025	0.61 (0.35, 1.06); 0.083
Hormonal contraceptive user	1.50 (0.89, 2.51); 0.125	
Government officer or private business owner	1.99 (1.22, 3.26); 0.006	1.66 (1.03, 2.68); 0.037
Any Breastfeeding		
Intention to breastfeed for at least 6 months postpartum	2.75 (1.70, 4.45); < 0.001	2.65 (1.65, 4.25); < 0.001
Initiation of breastfeeding within the first 24 h after delivery	1.51 (1.17, 1.96); 0.002	1.38 (1.08, 1.76); 0.009
Inversion or retraction of nipple	0.58 (0.34, 0.96); 0.036	0.68 (0.41, 1.10); 0.122
Hormonal contraceptive user	1.13 (0.86, 1.49); 0.369	

*RR* risk ratio, *CI* confidence interval

A *p*-value < 0.05 indicates statistical significance

## Discussion

This is the first study focusing on EBF in women with GDM through to 6 months postpartum, and it is also the first study to investigate breastfeeding in Thai women with GDM. The prevalence of EBF in women with GDM in this study was approximately 23% at 6 months after delivery, which was comparable to the prevalence of EBF in the general Thai population from a national report (23.1%) [8]. However, it was lower than the 2017–2021 national target of 50% and lower than the 34.8% prevalence from a global report published by the WHO [9]. Despite the known importance of EBF, few studies had reported the prevalence of EBF at 6 months postpartum in the general Thai population [8, 16, 17, 20]. The prevalence rates reported from previous Thai studies were lower than our result. A previous study reported an EBF rate of 23.8% at 3 months in mothers with GDM [21], which was lower than our result (35.9% at 3 months). The rate of any breastfeeding in the present study was 49.8% at 6 months postpartum which was similar to the rate observed in the general Thai population [20]. However, the rate of any breastfeeding in mothers with GDM differed among studies (31.3 to 65.7%), due to variations in breastfeeding definitions and assessment durations among studies [15, 22, 23].

Importantly, maternal intention to breastfeed until 6 months of infant age was an independent predictor of 6 months EBF and any breastfeeding practice. Other studies also reported a woman’s intention to breastfeed as being an important factor for optimizing breastfeeding practice in mothers with and without GDM [20, 24–26]. This finding suggests that a mother’s determination and willingness to breastfeed significantly influences breastfeeding duration. At the individual level, maternal intention to breastfeed was one of the important determinants of breastfeeding and the most commonly cited reasons for this intention was either personal norms or benefit of breastfeeding [27]. Therefore, health-care providers should support feeding decision and emphasize the benefits of breastfeeding at key moments during the antenatal and early postpartum period in order to enhance maternal motivation to maintain EBF for a minimum of 6 months after delivery and continued breastfeeding thereafter [27].

Maternal occupation was another factor that showed significant association with EBF for 6 months postpartum in this study. Some previous studies found a negative effect of working outside the home on EBF rate among working mothers [28, 29]. The effect of work on breastfeeding cessation may be due to either work that disallows or makes EBF difficult or work that has a lack of privacy or that limits the time needed to express breast milk. Availability or sufficiency of time to breastfeed or to pump breast milk may be a potential contributing factor that influences the ability to continue breastfeeding after the end of maternity leave. In the present study, we found being a government officer or a private business owner to be an independent predictor of 6 months EBF. This may be explained by the flexibility that a private business owner often has compared to one who works for others. In Thailand, mothers working in the government sector receive support from the government such as paid maternity leave and various supporting system for nursing mothers at work as well as breastfeeding breaking time and space for breast pumping. In addition, we found breastfeeding initiation within the first 24 h after delivery to be significantly associated with any breastfeeding for 6 months postpartum. The infants of mothers with GDM may have higher rates of complications, such as hypoglycemia and prematurity, and these conditions can result in prolonged separation of the mother and infant after delivery, delayed bonding, and increased likelihood of a need for supplementation with infant formula during the hospital stay. All of the aforementioned factors can have a negative effect on the initiation and continuation of breastfeeding. Moreover, based on our hospital policy, all infants of mothers with GDM were admitted to the nursery ward immediately after birth to monitor blood glucose level and to receive infant formula in order to prevent hypoglycemia, which can lead to delayed breastfeeding initiation. This practice may need to be reconsidered because mother-infant separation and prelacteal feeds are important barriers to timely breastfeeding initiation and ongoing breastfeeding in women with GDM who deliver at our hospital. The other possible explanation for the impact of delayed initiation on ongoing breastfeeding could be that women with GDM have a higher probability of developing delayed stage II lactogenesis [30]. Maternal overweight or obesity are also reported to be risk factors for delayed stage II lactogenesis [9, 31] due to a decreased prolactin response to infant suckling [32], which may cause breastfeeding difficulty and a lower rate of breastfeeding continuation. Thus, improved support from healthcare providers to enhance early initiation of breastfeeding, decrease mother and infant separation time, promote early skin-to-skin contact, and allow rooming-in after resolution of hypoglycemia may improve the breastfeeding initiation and continuation rate.

The strength of this study is that it is one of the few studies that provide information concerning the prevalence of breastfeeding and the factors associated with breastfeeding in women with GDM. Furthermore, we followed the breastfeeding behavior of mothers with GDM up to 6 months postpartum, which is the recommended duration of EBF. Another notable strength is the use of WHO definitions to define breastfeeding types. This study also has some mentionable limitations. First, there was no non-GDM control group to compare with mothers with GDM and there were a number of excluded women in our study. However, the size of our study population assured adequate statistic power to interpret the outcomes. Second, no data were collected from women with GDM relative to their attitudes toward or their knowledge about breastfeeding. We acknowledge that both of these factors could influence breastfeeding behavior. Third, we collected data concerning the amount of breastfeeding relative to the definitions of the different breastfeeding types, but data relating to the modes of feeding were not collected.

## Conclusion

The prevalence of EBF at 6-months postpartum was low among Thai mothers with GDM and was lower than the national and global target goal. Maternal intention to breastfeed for 6 months and breastfeeding initiation within 24-h postpartum were important factors associated with 6 months of breastfeeding. Health care providers should provide breastfeeding education, support feeding decision and help mothers to initiate breastfeeding in that might be improving breastfeeding rate. Hospital practice causing mother-infant separation is an important barrier to breastfeeding initiation and needs to be revised.

## Data Availability

The datasets used and/or analyzed during the current study are available from the corresponding author on reasonable request.
